# Ultraviolet Radiation Biological and Medical Implications

**DOI:** 10.3390/cimb46030126

**Published:** 2024-02-29

**Authors:** Tarek Al-Sadek, Nabiha Yusuf

**Affiliations:** Department of Dermatology, UAB Heersink School of Medicine, Birmingham, AL 35294, USA; talsadek@uab.edu

**Keywords:** ultraviolet radiation, UVA radiation, UVB radiation, melanoma, squamous cell carcinoma, basal cell carcinoma, cyclobutane dimers, 6-4 photoproducts, ROS

## Abstract

Ultraviolet (UV) radiation plays a crucial role in the development of melanoma and non-melanoma skin cancers. The types of UV radiation are differentiated by wavelength: UVA (315 to 400 nm), UVB (280 to 320 nm), and UVC (100 to 280 nm). UV radiation can cause direct DNA damage in the forms of cyclobutane pyrimidine dimers (CPDs) and 6-4 photoproducts (6-4PPs). In addition, UV radiation can also cause DNA damage indirectly through photosensitization reactions caused by reactive oxygen species (ROS), which manifest as 8-hydroxy-2′-deoxyguanine (8-OHdG). Both direct and indirect DNA damage can lead to mutations in genes that promote the development of skin cancers. The development of melanoma is largely influenced by the signaling of the melanocortin one receptor (MC1R), which plays an essential role in the synthesis of melanin in the skin. UV-induced mutations in the BRAF and NRAS genes are also significant risk factors in melanoma development. UV radiation plays a significant role in basal cell carcinoma (BCC) development by causing mutations in the Hedgehog (Hh) pathway, which dysregulates cell proliferation and survival. UV radiation can also induce the development of squamous cell carcinoma via mutations in the TP53 gene and upregulation of MMPs in the stroma layer of the skin.

## 1. Introduction

Ultraviolet (UV) radiation is an environmental stress factor that extensively affects the skin. Persistent UV exposure over extended periods contributes to various adverse events that weaken our immune suppression, accelerate photoaging, and increase our susceptibility to developing skin cancer. In this review, we will discuss UV radiation’s effects on our molecular biology, specifically on the types of DNA lesions, including cyclobutane pyrimidine dimers (CPDs) and 6-4 photoproducts (6-4PPs). Apart from direct damage from UV radiation, we will discuss the implications of indirect DNA damage through UV-induced reactive oxygen species (ROS). Understanding the etiology and biological implications remains pivotal in treating melanoma, basal cell carcinoma, and squamous cell carcinoma. Additionally, we will discuss the current treatment methods for melanoma, basal cell carcinoma, and squamous cell carcinoma.

## 2. Sources of Ultraviolet Radiation

Ultraviolet radiation (UVR) is in a distinct portion of the electromagnetic spectrum, ranging from 200 to 400 nanometers (nm), placing it just beneath the visible light range of 400 to 700 nm. UVR is primarily sourced from the sun and is differentiated into three categories based on wavelength: UVA (315 to 400 nm), UVB (280 to 320 nm), and UVC (100 to 280 nm) [1]. Alongside natural sources, artificial sources of UV radiation are becoming more common in dermatological conditions. Such sources include tanning beds and various therapeutic medical devices capable of emitting UVA, UVB, or UVC radiation. Industrial equipment, such as welding torches, also serves as a considerable artificial source of potent UV radiation [2]. The assortment of natural and artificial UV sources enriches the spectrum of UV exposure encountered in different environments, which potentially results in an elevated risk of skin cancer.

### 2.1. Ozone Layer

The stratosphere’s ozone layer serves as a critical barrier, absorbing much of the sun’s UV radiation and thereby shielding the Earth’s surface from its harmful effects [3]. In regions near the equator, the ozone layer is thinner; these areas experience higher levels of UV radiation, which correlates with increased rates of skin cancer at decreasing latitude [4]. Conversely, the ozone layer is thicker near higher latitudes, providing greater protection against UV radiation. In the last century, human activities, notably the emission of chlorofluorocarbons (CFCs) and other ozone-depleting substances (ODSs), have significantly weakened the ozone layer, increasing the risk of UV radiation reaching the Earth. The Montreal Protocol, established in the 1980s, has played a pivotal role in halting the depletion of the ozone layer by eliminating 99% of ODS emissions through bans on various applications, such as freon refrigerators. As a result of the Montreal Protocol, the ozone layer is anticipated to return to its baseline state by the mid-21st century [3]. Projections indicate a decrease in UV radiation between 30 and 60 degrees latitude by 2 to 5% in the north and 4 to 6% in the south, reflecting the positive impact of the protocol [5]. However, challenges remain due to rising greenhouse gas emissions, contributing to variability in the ozone layer and influencing UV radiation levels. Factors such as cloud cover, aerosols, and surface reflectivity are becoming more significant in determining UV exposure outside polar regions. Climate change, marked by increasing temperatures, further modifies UV radiation exposure, with research suggesting that the carcinogenic potential of UV radiation could increase by 5% with each degree Celsius rise in temperature [6]. This complex interplay of environmental changes highlights the ongoing need to monitor and adapt our strategies for protecting public health against the effects of UV radiation.

#### 2.1.1. Melanoma

The incidence of melanoma is on the rise in many developed countries, though the rates differ across various populations and age groups. Specifically, in countries like Canada, Italy, Norway, France, and Lithuania, there has been a notable increase in melanoma cases among older age groups. Conversely, younger demographics have seen a decline or stabilization in melanoma incidence [3]. This difference in trend between older and younger groups can largely be explained by the cumulative effect of UV radiation exposure over the years in older populations, including UV exposure prior to the implementation of the Montreal Protocol [7]. For younger individuals, the reduced incidence of melanoma is likely due to heightened awareness and use of sun protection measures [3]. Additionally, shifts in occupational trends toward more indoor jobs and preferences for indoor leisure activities have contributed to lower UV exposure among this group. These trends highlight the importance of continued public health efforts to promote sun safety and the impact of global environmental policies on public health outcomes.

#### 2.1.2. Squamous Cell Carcinoma and Basal Cell Carcinoma

Squamous cell carcinoma and basal cell carcinoma, collectively known as keratinocyte skin cancers (KCs), have seen varying trends in incidence across different regions. From 2005 to 2019, Australia, New Zealand, Iceland, and the United Kingdom reported an increased incidence of KCs, while the United States experienced relatively stable rates [3]. This disparity in KC incidence rates can be linked to a variety of factors, including differences in health policies, public awareness, accessibility to healthcare services, practices in reporting, and, potentially, genetic predispositions affecting skin cancer susceptibility. A significant aspect influencing the risk of developing KCs is exposure to ambient UV radiation, with populations in regions with high UV exposure facing a substantially increased risk. For instance, the lifetime risk of developing KCs in Australia is 3.5 times higher than in the United States [3]. This data implies that geographic areas subjected to more intense and consistent UV radiation exhibit higher incidences of KCs, underlining the critical impact of environmental factors on the prevalence of skin cancers.

## 3. Types of Ultraviolet Radiation

### 3.1. UVA Radiation

UVA rays have the longest wavelength within the ultraviolet spectrum, extending from 320 to 400 nanometers (nm), and constitute the majority—between 90% and 95%—of UV radiation that reaches the Earth’s surface. These rays are known for their ability to penetrate the skin deeply, reaching beyond the epidermis to the lower layers of the dermis. UVA radiation adversely impacts both epidermal keratinocytes and dermal fibroblasts, leading to long-term skin structure and function changes. The penetration of UVA radiation is significant, about 100-fold more than UVB radiation that can infiltrate the skin [1]. Despite their ability to reach deeper into the skin, UVA rays are generally less directly involved in skin carcinogenesis than UVB rays due to their relatively lower absorption by DNA [8]. The mechanism in which UVA rays cause DNA damage is primarily through indirect means, such as photosensitization reactions, which lead to the formation of 8-hydroxy-2′-deoxyguanine (8-OHdG) [9]. When skin chromophores absorb UVA rays, this interaction prompts the formation of reactive oxygen species (ROS), which can cause oxidative stress within the epidermal and dermal layers of the skin [10]. This oxidative stress is a key player in the pathogenesis of photoaging. It activates pathways like mitogen-activated protein kinases (MAPKs) and nuclear factor-kappa B (NF-κB), which elevate the levels of matrix metalloproteinases (MMPs) in the skin. MMPs are enzymes that break down structural proteins such as collagen and elastin. These proteins are crucial for maintaining skin elasticity and integrity [11]. Consequently, the breakdown of structural proteins by MMP contributes to the development of wrinkles and other signs of aging skin. Moreover, the use of tanning beds, which predominantly emit UVA radiation, has been linked to increased risks of erythema (skin reddening) and melanoma. Studies have shown that UVA radiation is responsible for 50% to 80% of the erythema associated with tanning beds, underscoring the potential risks associated with artificial UVA exposure [12] rather than the potential risks associated with UVB radiation exposure.

### 3.2. UVB Radiation

UVB rays have a wavelength ranging from 280 to 320 nm and are the primary cause of carcinogenic damage found in the skin. Despite comprising only 1% to 10% of sunlight that reaches our planet, UVB rays are the primary cause of overt skin damage, such as sunburns [13]. These rays primarily target the outermost skin layer, the epidermis, but have the capacity to penetrate the upper dermis [1]. For UVB rays to initiate a biological response, they must be absorbed by cellular molecules capable of transforming light energy into chemical signals. Genomic DNA is the primary absorber of UVB, and its interaction with UVB often leads to the formation of thymine dimers, which are distinctive indicators of UVB damage [1]. This results in the formation of cyclobutane pyrimidine dimers (CPDs) and 6-4 photoproducts (6-4PPs), which can accumulate and, if not properly repaired by the nucleotide excision repair (NER) system, significantly heighten the risk of skin cancer [14]. UVB exposure also has the potential to generate reactive oxygen species (ROS) via nicotinamide adenine dinucleotide phosphate hydrogen (NADPH) oxidase and cyclooxygenase (COX) enzymes in keratinocytes.

### 3.3. UVC Radiation

UVC has a wavelength between 100 and 280 nm, representing the most deleterious form of UV radiation due to its high energy [1]. Fortunately, UVC rays are completely filtered by the Earth’s ozone layer and never reach the ground.

## 4. Forms of DNA Damage

**Figure 1:**
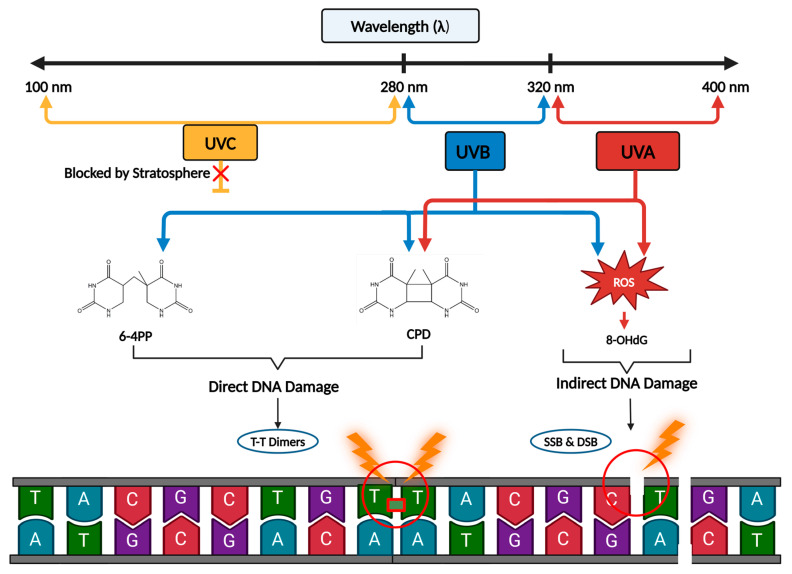
Types of UV-induced DNA damage. UVC rays have a wavelength between 100 and 280 nm, UVB rays have a wavelength between 280 and 320 nm, and UVA rays have a wavelength between 320 and 400 nm. UVC rays, the most mutagenic form of UV radiation, do not penetrate the stratosphere and, therefore, cannot induce DNA damage. In contrast, UVA and UVB rays can penetrate through the stratosphere and cause UV damage to the skin. The type of DNA damage depends on the type and dosage of UV radiation. UVB rays predominately cause direct DNA damage through the formation of 6-4 photoproducts (6-4PP) and cyclobutane pyrimidine dimers (CPDs) and cause indirect DNA damage through the formation of reactive oxygen species (ROS). UVA rays can only cause direct DNA damage by forming CPDs, not through 6-4PPs. UVA rays predominately cause indirect DNA damage by generating ROS and 8-hydroxy-2′deoxyguanine (8-OHdG). Both forms of direct DNA damage manifest primarily as thymine–thymine dimers in the DNA. Indirect DNA damage is caused by the formation of ROS, which can oxidize nucleotide base pairs, forming 8-OHdG and inducing single-stranded breaks (SSBs) and double-stranded breaks (DSBs) in the DNA.

The generation of photoproducts in the skin varies based on the type and amount of UV radiation exposure. Cyclobutane pyrimidine dimers (CPDs) and 6-4PPs are predominantly a result of UVB radiation (Figure 1). In contrast, 8-hydroxy-2-deoxyguanine (8-OHdG) is a prevalent marker for assessing DNA damage attributable to UVA radiation [1].

### 4.1. Cyclobutane Pyrimidine Dimers (CPDs)

Cyclobutane pyrimidine dimers (CPDs) are DNA lesions caused by adjacent pyrimidine bases becoming covalently bonded via a [2 + 2] cycloaddition reaction at the C5 and C6 carbon atoms [15]. This reaction can produce four types of dimers: T-T, T-C, C-T, and C-C CPDs. The formation of photoproducts is influenced by the GC% content in DNA, with a lower GC content favoring the production of T-T photoproducts and a higher GC content leading to less T-T photoproducts. Among these, T-T CPDs are the most commonly formed photoproduct, followed by T-C, C-T, and C-C CPDs in decreasing frequency [16]. Furthermore, CPDs are the most common form of DNA damage resulting from UV radiation, commonly associated with UVB radiation. While traditionally linked to UVB, research has shown that UVA rays can also lead to CPDs. UVA-induced CPDs are typically found at thymine–thymine sites and are induced through photosensitization [17]. This process is different from the direct absorption of UV rays by DNA, which is more typical with UVB-induced CPDs. Additionally, CPDs caused by UVA are considered more carcinogenic due to their ability to remain in the skin for longer durations and show increased reactivity at certain DNA sites [17]. This extended presence and heightened reactivity of UVA-induced CPDs highlight a distinct mechanism of skin damage that differs from the more direct DNA absorption associated with UVB rays.

### 4.2. Dark CPDs

UVA and UVB radiation can also contribute to the formation of ‘Dark CPDs’ by generating ROS and reactive nitrogen species (RNS). These molecules are transformed into a molecule called peroxynitrate, which reacts with melanin fragments to form dioxetanes. These dioxetanes then break down into two carbonyls, one of which is an excited-state triplet carbonyl with energy equivalent to a UV photon [18]. These excited-state triplet carbonyls can induce the formation of Dark CPDs in DNA. Interestingly, Dark CPDs are formed hours later after UV exposure, reaching peak levels 24 h post-exposure. Furthermore, Dark CPDs account for over half of all CPDs in melanocytes and keratinocytes post-UVR exposure [18]. Despite this, traditional sunscreens are not very effective against Dark CPDs. Recent research has highlighted the efficacy of Fernblock, an aqueous *Polypodium leucotomos* extract, in significantly reducing the formation of Dark CPDs due to its antioxidant properties [19].

### 4.3. 6-4 Photoproducts (6-4PPs)

The 6-4 photoproduct is another consequential UV-induced DNA lesion caused by UVB and UVA radiation. 6-4 photoproducts (6-4PPs) arise from a noncyclic bond between the C6 atom of one pyrimidine and the C4 atom of an adjacent pyrimidine, predominantly occurring as T-C 6-4PPs, with T-T 6-4PPs being less common [16]. Although they occur less frequently than CPDs, 6-4PPs are highly mutagenic because they significantly distort the DNA helix, contributing to the risk of UV-induced skin cancers [20]. Without proper repair by systems such as NER, these lesions can substantially increase the potential for skin cancer development.

### 4.4. Repair Mechanisms of CPDs and 6-4PPs

The synthesis of pyrimidine dimers such as CPDs and 6-4PPs upon UV radiation exposure significantly influences the development and progression of melanoma and non-melanoma skin cancers. These lesions are typically rectified by a suite of DNA repair mechanisms, including nucleotide excision repair (NER), base excision repair (BER), mismatch repair (MMR), and DNA double-strand break (DSB) repair [21]. NER is critical for rectifying CPDs and 6-4PPs and plays a vital role in melanoma prevention. The repair efficiency of CPDs varies, with the sequence C-T > C-C > T-C > T-T, indicating T-T dimers as the most resistant to repair by the NER system. This difference in repairability means that the longevity of photoproducts in human skin can vary significantly, with T-T dimers exhibiting the greatest resistance to repair mechanisms [16]. Furthermore, exposure to UVA radiation detrimentally affects the repair rate of CPDs more than UVB radiation, emphasizing the significant role of the type of UV radiation in DNA repair dynamics [16]. 

NER functions via two main pathways: global genomic repair (GG-NER), which addresses genome-wide damage, and transcription-coupled repair (TC-NER), which focuses on repairing damage on actively transcribed genes [22]. Both pathways are key defenses against UV-induced cancer. Individuals with conditions like xeroderma pigmentosum (XP), who lack functional NER, are significantly more prone to skin cancer due to inadequate UV damage repair. The NER pathway is made up of seven different genes from XP-A to XP-G, each of which plays a distinct role in the NER pathways. Patients with XP are classified according to which gene is mutated, with the addition of the XP variant (XP-V). The XP-V group exhibits normal NER function but lacks DNA polymerase (Pol η) due to a mutation in the POLH gene, which is involved in replicating damaged DNA [23]. Patients with mutations in XP-A, XP-B, XP-D, XP-F, and XP-G genes often endure more severe sunburns during adolescence, reflecting their heightened sensitivity to UV radiation. On the other hand, individuals with normally functioning transcription-coupled nucleotide excision repair (TC-NER) mechanisms, specifically those with XP-C, XP-E, and XP-V mutations, typically exhibit normal reactions to sunburn [23]. Interestingly, despite experiencing less severe immediate effects from sun exposure, the XP-C, XP-E, and XP-V groups are at a higher risk of developing skin cancer than the other XP groups.

#### Photolyases

Photolyases are a class of flavoproteins capable of repairing UVB-induced DNA damage, such as CPDs and 6-4PPs, through the absorption of blue light [24]. Interestingly, while photolyases are widespread across various organisms, including bacteria, fungi, plants, and most vertebrates, they are notably absent in mammals, including humans. This absence is attributed to evolutionary processes wherein mammals lost the photolyase gene. Consequently, humans and other mammals must rely on the nucleotide excision repair (NER) system to address UV-induced DNA damage. The NER process is complex and less efficient than the direct reversal mechanism employed by photolyases. Fortunately, scientific advancements have enabled the use of photolyase in topical applications, such as sunscreens and after-sun products [24]. By incorporating photolyase into these products, we can simulate its DNA repair benefits on human skin, offering enhanced protection against the harmful effects of UV radiation. This innovative approach marries evolutionary biology with biotechnology, providing a sophisticated method to shield our skin from UV-induced damage and potentially reduce skin cancer risk [25]. Research using transgenic mice expressing CPD-photolyase and 6-4PP-photolyase has shown a marked resistance to UV-induced skin cancer [26]. These findings highlight the protective effects of photolyase enzymes against UV radiation damage, offering promising insights into potential treatments for enhancing UV resistance and reducing skin cancer risk. However, obstacles such as high costs and the instability of protein extracts have limited its widespread adoption in sunscreen formulations. Despite these challenges, recent developments in gene therapy present a promising avenue, especially for individuals with compromised DNA repair mechanisms, like those with xeroderma pigmentosum (XP). Incorporating DNA photolyase directly into the cells of such patients could offer substantial benefits, potentially lowering their heightened risk of cancer [27].

### 4.5. Indirect DNA Damage (ROS)

UV radiation can also induce DNA damage indirectly via the production of ROS, which activates pathways such as nuclear factor (NF)-kB, potentially leading to tumorigenesis. ROS primarily inflict DNA damage by oxidizing nucleotide bases, notably converting guanine to thymine, producing 8-hydroxy-2′-deoxyguanine (8-OHdG) [15]. This oxidized base has a higher affinity for adenine than cytosine, prompting guanine-cytosine to adenosine-thymine mutations. Furthermore, ROS can induce the formation of single-stranded and double-stranded breaks in DNA and cause impairments to the topoisomerase complexes [15]. These mutations are predominantly repaired by BER, which plays a crucial role in fixing ROS-induced DNA damage. Compounds like glutathione (GSH) are also instrumental in neutralizing ROS.

UVB radiation significantly contributes to producing ROS in keratinocytes, leading to effects such as photoaging and apoptosis. Research by Beak et al. (2004) observed that UVB radiation increases ROS production in keratinocytes in a dose-dependent manner, primarily through the activity of NADPH oxidase and COX enzymes [28]. The buildup of these UV-induced ROS then leads to the activation of the NF-kB pathway, which significantly intensifies the apoptosis rate as a response to UVB radiation exposure [14]. Studies by Cavinato et al. (2017) have also shown that UVB exposure can induce the production of ROS, and cause inhibition of proteasome activity, which then triggers autophagy [29]. The deactivation of proteasomes caused by ROS generation and protein oxidation plays a significant role in the regulation of transcription factors involved in MMP-1 expression [30]. This sequence of events following UVB exposure provides a deeper insight into the multifaceted nature of UVB-induced damage.

#### Photoaging

A key aspect of the photoaging process is the reduced expression of dermal fibers, attributed to UV-induced ROS. Matrix metalloproteinases (MMPs), such as MMP-1, MMP-3, and MMP-9, play a crucial role in the degradation of extracellular matrix (ECM) proteins like collagen and elastin [11]. Their overproduction is particularly linked to photoaging, which leads to the development of wrinkles and loose skin. Dong et al. (2008) identified UVB-induced DNA damage to be the primary contributor to the upregulation of the MMP-1 gene in keratinocytes [31]. Moreover, UV-induced ROS are known to activate the mitogen-activated kinase family (MAPK) pathway, leading to the production of the activator-1 protein (AP-1), which oversees MMP transcription and inhibits transforming growth factor (TGF)-B signaling, which is crucial for synthesizing type I pro-collagen [32,33]. AP-1, along with NF-kB—another transcription factor induced by UV exposure—induce MMP expression in skin fibroblast. These processes demonstrate that AP-1 and NF-kB are central figures in the photoaging process.

### 4.6. Autophagy

Autophagy in skin cells plays an essential role in protection from UV damage. When skin cells are exposed to UV radiation, it can lead to the accumulation of toxic byproducts. Fortunately, autophagy can remove these toxic byproducts, thus reducing oxidative stress in cells. Typically, UV radiation can induce autophagy in epidermal keratinocytes, dermal fibroblasts, and melanocytes. The primary mechanism in which UVA exposure causes autophagy is linked to increased levels of oxidized phospholipids, oxysterols, and cholesterols in cells. The accumulation of these molecules acts as signals for the initiation of autophagy in epidermal keratinocytes and dermal fibroblasts, which helps manage oxidative stress induced by UVA radiation [34]. In addition, UVA radiation impairs the transcription of crucial autophagy genes such as p53, p62, and Sestrin2 (SESN2). These genes can stimulate autophagy by 5′ adenosine monophosphate-activated protein kinase (AMPK) signaling pathway [30]. Studies have indicated that UVA radiation can disrupt the autophagy process in fibroblasts, primarily through alterations in lysosomal functioning. Key aspects of this impaired autophagic process include a decrease in lysosomal acidity and a reduction in the expression of cathepsins B, L, and D [30]. While it is clear that excessive UVA exposure can cause impaired lysosomal function in fibroblasts, the precise mechanisms and implications of this disruption are still the subject of ongoing research. Moreover, excessive exposure to UVB radiation can induce apoptosis by creating sunburn cells, a phenomenon where keratinocytes undergo apoptosis. This process is initiated by UVB-induced DNA damage in keratinocytes, triggering the release of various inflammatory mediators, including cytokines such as interleukin (IL)-1α, IL-6, and tumor necrosis factor (TNF)-α. Additionally, chronic UVB radiation exposure can cause downregulation of autophagy in dermal fibroblast through inhibition of ultraviolet radiation resistance-associated gene (UVRAG) or activation of the PI3K/Akt pathway [30,34].

Moreover, if these damaged cells fail to undergo apoptosis, they risk turning cancerous. However, if apoptosis is triggered too frequently, it can deplete the basal keratinocytes, which are essential for normal skin regeneration. The balance between pro-apoptotic and anti-apoptotic factors determines whether a cell undergoes apoptosis or survives. Persistent UVB exposure can disrupt this balance by inducing mutations in crucial genes that regulate cell growth and survival, like proto-oncogenes or tumor suppressor genes [14]. Mutations in the p53 gene, vital in the cell’s defense against DNA damage, are commonly found in sun-exposed skin, though only a fraction of such mutated cells, like those in actinic keratoses, progress to cancer [14].

## 5. Melanoma

Chronic exposure to UV radiation is a significant risk factor in the development of melanoma, contributing to around 60% to 70% of melanoma cases [35]. The impact of UVB radiation in the development of melanoma is well-documented, in contrast to the less clear role of UVA radiation. Studies using mouse models have identified UVB radiation as a primary factor in initiating the development of melanoma. On the other hand, UVA radiation is less potent in inducing melanoma, with its effects observed mainly in specific strains of pigmented mice [36]. Furthermore, a long history of childhood remains a significant risk factor for developing melanoma in later years [36]. Moreover, sunbeds, which emit primarily UVA rays, have also been associated with a heightened risk of melanoma [37].

### 5.1. MC1R

Skin complexion plays a significant role in influencing melanoma risk, primarily through the types of melanin produced: eumelanin and pheomelanin. Each type of melanin has distinct roles in both the development and prevention of melanoma. Eumelanin, the darker pigment more abundant in individuals with darker skin tones, offers protective benefits against UV radiation. This protective effect is attributed to the presence of DHICAs in eumelanin’s structure, providing a natural defense against UV-induced damage. In contrast, pheomelanin, a lighter pigment found more commonly in people with fairer complexions, is more susceptible to UV damage than eumelanin, primarily through the generation of ROS [9]. This vulnerability to UV radiation makes individuals with high pheomelanin and low eumelanin levels more prone to melanoma.

The melanocortin 1 receptor (MC1R) plays a central role in skin pigmentation, as it regulates the type of melanin synthesized. When a-melanocyte-stimulating hormone (αMSH) binds to MC1R, it leads to the production of eumelanin, enhancing the skin’s defense against UVR-induced damage and increasing NER activity. In melanocytes, UV radiation stimulates αMSH and MC1R signaling. Active MC1R signaling and the resulting higher levels of cAMP lead to increased melanin production, making cAMP levels a potential therapeutic target for treating individuals with MC1R-inactivating mutations [35]. Additionally, αMSH treatment has also been shown to be effective against UV-induced damage by improving DNA repair. However, diminished or inactivated MC1R signaling results in a higher pheomelanin-to-eumelanin ratio. Individuals with higher levels of pheomelanin face a greater risk of developing melanoma compared to those with higher eumelanin levels [9]. This increased risk highlights the importance of eumelanin synthesis in the protection of UV damage in the skin.

#### BRAF

In a substantial number of melanoma cases, estimated to be around 40% to 50%, activating mutations are observed in the BRAF gene [38]. This proto-oncogene gene encodes the serine-threonine protein kinase B-RAF, a crucial component of the MAPK (RAS-RAF-MEK-ERK) signaling pathway. Typically, BRAF is involved in either homo- or heterodimerization with another RAF kinase in response to growth signals, playing a vital role in regulating cellular growth and proliferation [39]. However, mutations in the BRAF gene, which substitutes valine with glutamic acid, can result in a 480-fold increase in BRAF kinase activation [40]. This hyperactivation results in continuous stimulation of the MAPK pathway, leading to unchecked cell proliferation and survival. Notably, the V600E mutation accounts for about 90% of all activating mutations in the BRAF gene [41]. These BRAF mutations, especially the V600E variant, are frequently found in various types of nevi. However, the presence of a BRAF mutation alone often does not lead to melanoma, as many nevi with this mutation tend to remain stable and do not progress to a malignant state [42]. This suggests that while the BRAF mutation is a crucial step, it is not solely decisive in the transition from a benign nevus to an aggressive melanoma. Additional genetic alterations or environmental factors may be necessary for a nevus with a BRAF mutation to transform into melanoma.

### 5.2. NRAS

The NRAS gene, like BRAF, is a crucial component of the MAPK pathway and is implicated in 15% to 20% of melanoma cases. In its typical state, NRAS functions as a GTPase, regulating the activity of RAF proteins within the MAPK pathway [43]. Ordinarily, NRAS remains in an inactive, GDP-bound state. However, when stimulated, NRAS swaps its GDP for GTP, transitioning to an active state that triggers various intracellular signaling pathways, including the MAPK pathway [44]. Mutations in the NRAS gene, especially the common Q61 mutation, impede the ability of NRAS to revert from its active GTP-bound form to the inactive GDP-bound state [44]. As a result, NRAS becomes perpetually active, leading to continuous MAPK pathway activation. This then stimulates uncontrolled cellular growth, proliferation, and survival, which significantly increases the risk of melanoma development. Despite both NRAS and BRAF mutations being fairly common in the development of melanoma, rarely do both mutations occur in the same patient [45]. This rarity suggests that a single activating mutation in either NRAS or BRAF is typically sufficient to catalyze the pathway’s activity toward tumor development.

### 5.3. Alternate Autocrine

In cases of melanoma where neither BRAF nor RAS gene mutations are present, the MAPK pathway can still become activated through alternative autocrine mechanisms. One example involves the overexpression of c-mesenchymal-epithelial transition factor (c-Met), a receptor tyrosine kinase that binds to hepatocyte growth factor and subsequently triggers MAPK signaling [46]. Additionally, the pathway can be activated by downregulating proteins that usually serve as inhibitors of the MAPK pathway. Such inhibitors include RAF-1 inhibitory protein and Sprouty-2 (SPRY-2), which are crucial in maintaining the control of MAPK signaling under standard physiological conditions [47].

### 5.4. Diagnosis

The TNM staging system is an essential tool used to categorize the stage of skin cancer based on tumor size (T), lymph node involvement (N), and the presence of metastasis (M). Stage I represents localized melanoma with no evidence of metastasis. Stage II represents melanoma with an increased risk of recurrence. Stage III indicates that melanoma has spread to nearby lymph nodes. Stage IV, the most advanced, indicates that cancer has spread to distant parts of the body, requiring comprehensive systemic therapy [48]. The primary method for diagnosing superficial melanoma involves a visual skin examination using the ACBDE (asymmetry, border irregularity, color variegation, diameter > 6 mm, evolution) checklist. The use of a dermoscope, a tool that essentially combines a magnifying glass with a powerful light, is an important diagnostic tool used to identify characteristics of melanoma not visible to the naked eye with a sensitivity ranging from 76–92% [49,50]. In addition, the “ugly duckling” sign is a standard method used to evaluate suspicious lesions by comparing the presence of a single lesion with the patient’s nevus phenotype [49]. Following the identification of suspicious lesions, a biopsy by local excision should be performed to confirm a diagnosis. Furthermore, sentinel lymph node biopsy (SLNB) is a minimally invasive surgical procedure recommended for patients with intermediate-thickness melanomas to determine if the cancer has spread to nearby lymph nodes [51]. In addition, reflectance confocal microscopy is an imaging tool used to examine cells and tissues of the epidermis and papillary dermis lesions [52]. Moreover, the landscape of skin cancer screening is rapidly evolving due to recent advances in artificial intelligence (AI), with machine learning (ML) and deep learning (DL) algorithms at the forefront. These technologies can analyze large datasets, learning to identify patterns and features indicative of skin cancer that may not be apparent to the human eye. A notable milestone in this area is the FDA’s clearance of DermaSensor’s AI-powered tool for skin cancer detection [53]. This device utilizes elastic scattering spectroscopy (ESS) to differentiate between normal and abnormal tissue in vivo, offering a non-invasive approach. In clinical trials, the DermaSensor demonstrated a remarkable sensitivity rate of 95.5% in identifying skin cancers, surpassing the detection rates of primary care physicians, which stand at around 83% [54]. This development marks a significant step forward in the potential for AI to enhance the accuracy and reliability of skin cancer diagnoses.

### 5.5. Prognosis

Melanoma is recognized as the most lethal type of skin cancer, accounting for 80% of skin cancer-related deaths [55]. The 5-year relative survival rate of melanoma is 93.5%, whereas squamous cell carcinoma and basal cell carcinoma have a 5-year survival rate of 90% and 100%, respectively, as shown in Table 1 [56,57,58]. The prognostic significance of melanoma thickness in survival outcomes has been extensively documented across various studies, illustrating a relationship between tumor depth and long-term patient survival. Research demonstrates that melanoma-specific survival rates significantly decrease as Breslow thickness increases. For instance, a population-based study revealed a 20-year melanoma-specific survival rate of 96%, with rates decreasing from 98.3% for tumors < 0.25 mm in thickness to 89.0% for those 0.75 to 1 mm thick [59]. These findings underscore the critical threshold effect of tumor depth on survival, with a notable decline in prognosis observed as thickness approaches 0.75 mm. A single-institution analysis supported the adoption of a ≤0.8 mm cutoff for melanoma thickness, beyond which a significant decline in melanoma-specific survival rates was observed over both 10- and 20-year periods [60]. Collectively, these studies affirm the pivotal role of tumor thickness as a primary prognostic factor in melanoma. Moreover, despite a lower incidence of melanoma in Black populations compared to White populations, their 5-year relative survival rate is notably lower [61]. Black individuals more frequently present with melanoma at advanced stages, with a greater incidence of regionally advanced or distant disease. Black individuals had a significantly higher mortality risk than non-Hispanic White individuals [61]. This trend is similarly observed in the Hispanic American population, which, despite a lower incidence rate, faces a growing burden of the disease and is more likely to present with advanced stages of melanoma than their non-Hispanic White counterparts. This disparity can be attributed to several factors, including differences in the clinical manifestation of skin cancers among ethnicities, which can lead to challenges in early detection, particularly in pigmented skin. Additionally, disparities in patient education, the cultural relevance and competence of healthcare information, and access to dermatological care play significant roles. Other contributing risk factors include occupational exposure and HIV infection, which is linked to an elevated risk of developing melanoma [62]. 

### 5.6. Treatment

The field of melanoma treatment for advanced cases has undergone a significant transformation with the development of targeted therapies that specifically inhibit certain genetic changes within tumor cells. A major breakthrough in this area has been the introduction of molecule inhibitors that target mutations in the BRAF gene and other components of the MAPK/ERK pathway. These inhibitors have shown effectiveness in patients with melanoma, particularly those with the BRAF V600E mutation.

BRAF inhibitors like vemurafenib, encorafenib, and dabrafenib have emerged as effective agents for BRAF monomers. In patients with metastatic melanoma carrying the BRAF V600E mutation, these drugs have become instrumental in inducing tumor regression and prolonging survival in patients. However, BRAF inhibitors are limited because the development of resistance mechanisms often leads to the reactivation of the MAPK pathway through alternative routes. Fortunately, combining BRAF inhibitors with MEK inhibitors has proven effective by reducing the risk of disease progression by 25% and increasing response rates [63].

MEK inhibitors such as trametinib, cobimetinib, binimetinib, and selumetinib have demonstrated promising outcomes in treating melanomas containing BRAF mutations [64]. These medications work downstream from RAS and RAF activation in the MAPK pathway. Furthermore, the effectiveness of MEK inhibitors is not restricted to BRAF mutant melanomas but also extends its efficacy in treating uveal melanoma, characterized by mutations in the GNAQ or GNA11 gene [65].

Checkpoint inhibitor immunotherapy stands out as an effective systemic approach for managing advanced melanoma. Treatments that employ programmed cell death (PD-1) inhibitors, including pembrolizumab and nivolumab, have been advantageous, enhancing patient survival outcomes. While PD-1 inhibitors are effective as standalone therapies, their combination with cytotoxic T lymphocyte-associated protein 4 (CTLA-4) inhibitors, such as ipilimumab, has significantly boosted survival rates beyond what PD-1 monotherapy achieves [66]. In a 5-year clinical trial, overall survival rates at the 5-year mark for the group receiving the combination of nivolumab and ipilimumab had a survival rate of 52%. In comparison, those on nivolumab alone had a rate of 44%, and the ipilimumab group had a rate of 26%. Notably, within the nivolumab-plus-ipilimumab group, patients with BRAF mutations had a higher five-year overall survival rate of 60%, compared to 48% for those without these mutations [66,67]. Additionally, novel therapeutic strategies have emerged, such as pairing a LAG-3 inhibitor like Relatlimab with nivolumab, offering another potent combination for improving treatment efficacy [68].

## 6. Basal Cell Carcinoma

Basal cell carcinoma (BCC) is the most common form of skin cancer. The primary risk factor for developing BCC is intense intermittent exposure to ultraviolet radiation (UVR). In addition to these environmental influences, genetic modifications significantly contribute to BCC pathogenesis. Specifically, mutations in the Hedgehog (Hh) signaling pathway play a vital role in the development of BCC. This pathway’s alteration underscores BCC’s multifaceted nature, involving external and internal factors in its onset and progression. Approximately 85% of sporadic BCC cases are caused by genetic mutations affecting crucial proteins in the Hh signaling pathway [69]. This pathway is instrumental during embryonic development for organ formation and patterning but also retains a vital role in adulthood, contributing to cell regeneration and maintaining bodily homeostasis. In the skin specifically, the Hh pathway is essential for regulating and developing stem cells in hair follicles and sebaceous glands.

### 6.1. Hedgehog Pathway

The Hedgehog (Hh) signaling pathway is intricately regulated, with the Ptch1 protein acting as its primary inhibitor in the absence of hedgehog ligands such as Sonic Hedgehog, Indian Hedgehog, or Desert Hedgehog. When these hedgehog ligands are present, they bind to and inhibit Ptch1, leading to a different cascade of events. This interaction results in the activation and buildup of the Smoothened (SMO) protein within the cilium, sparking a series of downstream signals that eventually activate GLI transcription factors [70]. These GLI factors, controlled by the Hh pathway, play a pivotal role in initiating the expression of genes vital for cell differentiation, proliferation, and survival. Their activity is central to the normal functioning of various cellular processes, particularly in skin health.

#### PTCH1

Loss-of-function mutations in the Patched-1 (PTCH1) gene are found in approximately 70% to 75% of basal cell carcinoma (BCC) cases [69,71,72]. The mutation patterns observed in these PTCH1 genes strongly suggest a connection to UV-induced damage, as evidenced by typical UV signature mutations like C-T transitions or CC to TT double base changes [73,74]. These mutations are notably prevalent in sporadic cases of BCC, particularly among patients with xeroderma pigmentosum (XP). In addition to UV exposure, oxidative stress has also been shown to create similar mutation patterns in the PTCH1 gene [71]. On the other hand, 10% to 20% of BCC cases are characterized by gain-of-function mutations in the SMO gene [72]. These mutations lead to the heightened activation of the Hedgehog (Hh) signaling pathway, evidenced by the overexpression and increased activity of GLI proteins [75]. While the presence of mutations in both PTCH1 and SMO genes is central to the development of BCC, the precise mechanisms through which upregulation of the Hh pathway contributes to tumorigenesis remain complex. The prevailing view in academia is that the accumulation of GLI proteins is the primary driver in the development of BCC.

### 6.2. Basal Cell Nevus Syndrome

Basal cell nevus syndrome (BCNS), commonly known as Gorlin Syndrome, is an autosomal dominant genetic condition caused by a defective allele in the PTCH1 gene located on chromosome 9q [76]. Individuals with Gorlin Syndrome are significantly more susceptible to developing BCC. BCC’s pathogenesis in these patients is explained by the two-hit hypothesis, which posits that BCC development necessitates the inactivation of both alleles of the PTCH1 gene [76]. The second allele is typically deactivated due to environmental factors, particularly UV radiation. Additionally, the genetic scope of Gorlin Syndrome extends to mutations in other tumor suppressor genes, including the suppressor fused gene (SUFU) on chromosome 10q and PTCH2 on chromosome 1q [77]. Beyond genetic predisposition, having a family history of BCC or squamous cell carcinoma (SCC) also significantly increases the likelihood of developing BCC.

#### TP53

Apart from the Hh pathway, the TP53 tumor suppressor protein also plays a crucial role in the pathogenesis of BCC. Inactivating mutations in the TP53 gene are a frequent genetic alteration found in about 50% to 60% of sporadic BCC [78]. In approximately 30% to 50% of TP53 mutations exhibit “UV signature mutations” [73]. These patterns strongly suggest that UV radiation is the primary cause of mutations in the TP53 gene. This association is further supported by observations that TP53 mutations are less common in BCC patients who regularly use sunscreen than those who do not [79]. This finding highlights the role of UV radiation on genetic alterations in BCC and underscores the importance of using sunscreen in mitigating the risk of developing BCC. Furthermore, like mutations found in PTCH1 and SMO genes, mutations in TP53 can also lead to increased signaling in the Hh pathway, specifically through the upregulation of the SMO protein.

### 6.3. Diagnosis

The diagnosis of BCC involves a visual examination of the skin for manifestations of BCC lesions. Other diagnostic tools utilized for better diagnostic accuracy include dermoscopy. Following a diagnosis of BCC, a biopsy is performed to confirm the diagnosis and assess the risk of recurrence [80]. 

### 6.4. Treatment

The front-line approach to treating BCC involves surgical excision, primarily aimed at completely removing the tumor to reduce the likelihood of its recurrence. Standard excision (SE) is typically the method of choice depending on the specific characteristics of the tumor. In more severe or complex cases of BCC, Mohs micrographic surgery is preferred due to a higher success rate in complete tumor removal [81]. Photodynamic therapy is a minimally invasive procedure used to treat superficial BCC [81]. Additionally, pharmacological inhibitors targeting the Hedgehog (Hh) pathway have proven effective, particularly in treating BCC cases where surgery is not an option. Vismodegib and Sonidegib, both FDA-approved drugs, function as inhibitors of the SMO protein [82]. Administration of these drugs prevents the activation of GLI transcription factors, which has been shown to be effective in controlling difficult-to-treat BCCs when surgical treatments are not ideal. Other non-invasive treatment options include topical therapies such as 5-fluorouracil and imiquimod [81]. 

## 7. Squamous Cell Carcinoma

Cutaneous squamous cell carcinoma (SCC), the second most common type of non-melanoma skin cancer, is primarily caused by UV radiation exposure [83]. SCC primarily originates from the malignant transformation of keratinocytes in the epidermis [56]. A significant precursor to SCC is actinic keratosis (AK). AK is caused by UVR exposure and is marked by atypical changes in epidermal keratinocytes. Approximately 10% of AK cases eventually progress to SCC [84]. In addition to UVR exposure, other important risk factors for SCC include tobacco use, alcohol consumption, and exposure to human papillomavirus (HPV), particularly HPV-16 [85]. These factors collectively contribute to the risk and pathogenesis of SCC, highlighting the interplay of environmental, lifestyle, and genetic elements in its development.

### 7.1. HPV

SCC tumors exhibit different mutational profiles depending on their HPV status. HPV-positive SCC tumors are characterized by a distinct molecular pattern, including the loss of TNF receptor-associated factor 3 (TRAF3) and amplification of E2 promotor binding factor 1 (E2F1). In contrast, HPV-negative SCC tumors typically display mutations in CDKN2A and TP53 [85].

### 7.2. TP53

In the early stages of cutaneous SCC development, mutations in the TP53 gene play a pivotal role, contributing to 54% to 95% of SCC cases [56]. These genetic alterations are primarily induced by UV radiation exposure, as evidenced by “UV signatures” such as C-T and CC to TT transitions in DNA. Inactivation of the TP53 gene leads to a decrease in apoptosis and unchecked proliferation, contributing significantly to the onset and advancement of SCC.

### 7.3. MMP-2

Under normal conditions, the basement membrane prevents tumor invasion into the underlying connective tissue by acting as a barrier between the epidermis and the dermis. However, in cases of SCC and BCC, collagen IV is degraded in the basement membrane by MMP-2 and MMP-9. This results in the epithelial-mesenchymal transition of keratinocytes, which increases the invasive behavior of SCC [86]. Interestingly, the overexpression of MMP-2 in the stroma is exceptionally increased in SCC cases compared to BCC cases [87].

### 7.4. Diagnosis

The diagnosis of squamous cell carcinoma typically involves visual examination of the skin for any abnormal skin lesions that might indicate the presence of cancer. Dermoscopy and reflectance confocal microscopy (RCM) are non-invasive tools that aid in this examination, allowing for a closer look at skin lesions. High-frequency ultrasonography, another non-invasive technique, shows high diagnostic accuracy, with success rates ranging from 73% to 97% [88]. If these examinations suggest the presence of cancer, a biopsy is conducted to ascertain whether the lesion is in situ (Bowen’s disease) or an invasive squamous cell carcinoma. Advanced imaging technologies like computed tomography (CT) or magnetic resonance imaging (MRI) are used to assess the depth of invasion, providing valuable information for determining the appropriate treatment plan [88].

### 7.5. Prognosis

The prognosis for patients with primary cutaneous squamous cell carcinoma (cSCC) is generally positive, with a 5-year survival rate of 90% [57,82]. Cutaneous squamous cell carcinoma (SCC) poses a significant risk of metastasis, particularly when the tumor thickness exceeds 2.0 mm. The risk escalates dramatically for tumors thicker than 6.0 mm, where both metastasis and local recurrence become highly probable [89,90].

### 7.6. Treatment

Surgical excision remains the front-line treatment option for treating SCC, with a cure rate of approximately 90% [91]. For more high-risk SCC or immunocompromised patients, Mohs micrographic surgery is recommended for treatment [88]. Other treatment options for SCC include photodynamic therapy, topical therapies, such as 5-fluorouracil, and imiquimod [87]. Imiquimod is a toll-like receptor-7 agonist, which induces proinflammatory cytokines and promotes apoptosis of tumors [92]. Specifically, in patients with actinic keratosis treated with imiquimod, imiquimod has been shown to be an effective treatment option for reducing the risk of developing SCC [93,94].

## 8. Conclusions

Ultraviolet radiation (UVR) is a significant risk factor in the development of melanoma and non-melanoma skin cancer. Depending on the type of UV radiation and dosage, UV exposure can cause DNA lesions by forming CPDs, 6-4PPs, and ROS. These DNA lesions caused by UV exposure are intrinsically linked to the development of skin cancers, such as melanoma, basal cell carcinoma, and squamous cell carcinoma. UV exposure in melanoma development is highly implicated by mutations in the NRAS and BRAF genes and influenced by the amount of eumelanin produced in the skin. Basal cell carcinoma, the most common form of skin cancer, is primarily caused by UV-induced mutations of proteins involved in the Hedgehog pathway. Squamous cell carcinoma is primarily caused by UV-induced mutations in the TP53 gene and upregulation of MMPs, with factors like tobacco use, alcohol use, and HPV infections exacerbating SCC development.

Discoveries around the formation of cyclobutane pyrimidine dimers (CPDs), 6-4 photoproducts (6-4PPs), ‘Dark CPDs,’ and ROS-induced lesions due to UV exposure have been pivotal in developing innovative approaches to treatment and prevention. For example, Fernblock, a *Polypodium leucotomos* extract, has emerged as a potential substance for enhancing UV radiation resistance by minimizing the formation of Dark CPDs. Additionally, studies on photolyases, have uncovered that these enzymes have a more proficient DNA repair system than our native nucleotide excision repair (NER) system, highlighting the potential use of photolyases for sun protection.

Gene therapy offers a promising avenue for individuals with xeroderma pigmentosum (XP), in which patients could be cured by integrating transgenes that encode for specific XP genes or photolyases genes. This approach could significantly reduce the heightened skin cancer risk XP patients face. Moreover, artificial intelligence (AI), mainly through machine learning and deep learning algorithms, has recently revolutionized the field of skin cancer diagnosis. Recent studies have shown that AI is already considered a superior diagnostic tool to traditional visual examinations, positioning it as a likely primary method for skin cancer screening in the future. The FDA-approved DermaSensor, which employs elastic scattering spectroscopy (ESS) for detecting skin cancer, showcases the potential of AI to provide non-invasive, accurate diagnostics of skin cancer in real time.

As we look to the future, the evolution of more advanced AI algorithms is expected to improve skin cancer diagnosis accuracy dramatically. These algorithms could leverage multi-modal data, including genetic, phenotypic, and environmental information, to provide a holistic assessment of an individual’s risk for skin cancer.

Furthermore, the applications of gene therapy have the potential to directly eliminate genetic factors that predispose individuals to skin cancer, paving the way toward a future where genetic risk factors are no longer a determinant of skin cancer development. Overall, the merging of these cutting-edge technologies and cross-disciplinary research is set to alter traditional approaches to skin cancer, from prevention to treatment, ultimately aiming to significantly lessen the global impact of skin cancer.

## Figures and Tables

**Table 1:** Survival rates of skin cancer.

Type of Skin Cancer	5-Year Survival Rate
Melanoma	93.5%
Squamous Cell Carcinoma	90%
Basal Cell Carcinoma	100%

## Data Availability

All data presented can be accessed in the main text of this review article or on request from the corresponding author.
